# Insulin-like growth factor 2 receptor is a key immune-related gene that is correlated with a poor prognosis in patients with triple-negative breast cancer: A bioinformatics analysis

**DOI:** 10.3389/fonc.2022.871786

**Published:** 2022-10-18

**Authors:** Ying Zhong, Xinyu Ren, Xi Cao, Yali Xu, Yu Song, Yidong Zhou, Feng Mao, Songjie Shen, Zhe Wang, Qiang Sun

**Affiliations:** ^1^ Department of Breast Disease, Peking Union Medical College Hospital, Beijing, China; ^2^ Department of Pathology, Peking Union Medical College Hospital, Beijing, China

**Keywords:** poor prognosis, triple negative breast cancer, TIL (tumor infiltrating lymphocytes), CD8+ TILs, IGF2R

## Abstract

**Background:**

Immunotherapy plays an important role in the treatment of triple-negative breast cancer (TNBC). This study aimed to identify immune-related genes that are associated with the prognosis of patients with TNBC as possible targets of immunotherapy, alongside their related tumor-infiltrating lymphocytes (TILs).

**Methods:**

The clinical data and gene expression profiles of patients with breast cancer were extracted from The Cancer Genome Atlas (TCGA) and Gene Expression Omnibus (GEO) databases and divided into training (n = 1,053) and verification (n = 508) groups. CIBERSORT was used to predict the differences in immune cell infiltration in patient subsets that were stratified according to risk. Gene Ontology (GO) enrichment analysis was used to identify pathways associated with immune-related genes in patient subsets that were stratified according to risk. The clinical data and insulin-like growth factor 2 receptor (IGF2R) expression profiles of patients with breast cancer were extracted from METABRIC. The expression of IGF2R and TILs were evaluated in a cohort containing 282 untreated patients with TNBC. The correlations of IGF2R expression, TILs, and clinicopathological parameters with patient prognosis were analyzed in the whole cohort.

**Results:**

The prognostic model, which was composed of 26 immune-related gene pairs, significantly distinguished between high- and low-risk patients. Univariate and multivariate analyses indicated that the model was an independent prognostic factor for breast cancer. Among the identified genes, the expression of IGF2R significantly distinguished between high- and low-risk patients in TCGA (*P* = 0.008) and in METABRIC patients (*P <* 0.001). The expression of IGF2R was significantly associated with clinical risk factors such as TNBC, estrogen receptor (ER)–negative expression, human epidermal growth factor receptor 2 (HER2)–positive expression, and age ≤60 years old in METABRIC patients. In addition, the patients with IGF2R-positive expression had lower disease-free survival (DFS) rates than those with IGF2R-negative expression in the TNBC cohort (67.8% vs. 78.5%, *P* = 0.023). IGF2R expression also was significantly negatively correlated with TILs, particularly with CD8^+^ TILs and CD19^+^ TILs in the cohort of patients with TNBC.

**Conclusion:**

IGF2R can be used as an indicator of a poor prognosis in patients with TNBC and as a potential target and research direction for TNBC immunotherapy in the future.

## Introduction

Breast cancer is the most serious malignant tumor threatening the health of women worldwide. It is the leading global cause of cancer deaths in women and remains incurable when it reaches an advanced stage (1). Approximately 3%–10% of patients with new breast cancer are diagnosed with distant metastasis (2). Metastatic breast cancer remains an almost incurable disease, with an overall survival (OS) period of approximately 3 years and a 5-year survival rate of approximately 25% (3).

Insulin-like growth factor 2 receptor (IGF2R) is a membrane-binding glycoprotein whose main function is transporting lysosomes from the trans-Golgi network to the lysosomes. It plays an important role in cell growth and survival, and its expression is closely related to tumors (4). IGF2R is also associated with a variety of malignancies that include cervical cancer (5), bladder cancer (6), osteosarcoma (7), and mucosal melanoma (8). The loss of IGF2R activity affects tumor growth, apoptosis, angiogenesis, and invasion (9). IGF2R plays an important role in clearing apoptotic cells to maintain the stability of tissue environments (10). IGF2R has been confirmed to be involved in latent transforming growth factor beta (TGFβ) activation in human fibroblasts (11). In addition, TGFβ family cytokines are involved in immune regulation, extracellular matrix synthesis, as well as the proliferation, differentiation, and development regulation of various types of cells (12). Moreover, IGF2R plays a key role in the survival of CD8^+^ T cells (13) and in the activation and differentiation of T cells (14). Furthermore, some immunological mechanisms and pathways controlled by IGF2R have been discovered (15). However, the role of IGF2R in the immunotherapy of patients with breast cancer requires further study.

Cytotoxic chemotherapy, has long been the main treatment for triple-negative breast cancer (TNBC), and TNBC is more likely to metastasize than other types of breast cancer (16). The proportion of tumor-infiltrating lymphocytes (TILs) in TNBC is much greater than that in hormone receptor (HR)–tumors, and the increased proportion of TILs indicates a better prognosis (17). The cytotoxic T-lymphocyteassociatedantigen 4 (CTLA-4) and programmed death 1 (PD-1)/programmed death ligand 1 (PD-L1) have been observed to block TILs and to promote tumor growth and progression (18). Meanwhile, immune checkpoint inhibitors (ICIs) have been used successfully in the treatment of cancer (19), and immunotherapy has become the first treatment choice for patients with TNBC diagnosed with PD-L1–positive tumors (20). Anti-trophoblast cell surface antigen 2 is an antibody–drug conjugate that has been demonstrated to improve the progression-free survival of patients with metastatic TNBC (21). The poly-ADP ribose polymerase (PARP) inhibitors olaparib and talazoparib have been used in patients with TNBC with the mutant breast cancer gene *BRCA* who were resistant to chemotherapy (22). In addition, chimeric antigen receptor-positive T cells have been observed to kill tumor endothelial cells and tumor endothelial marker-8–positive TNBC cells by secreting immune-stimulating cytokines, but the relevant research is still in the preclinical stage 23. For patients with TNBC, the clinical benefit of immunotherapy is limited and remains in the research stage. Consequently, tumor immunity needs to be better understood to identify additional immune biomarkers and potential therapeutic targets.

To address these issues, in the present study, we aimed to identify powerful biomarkers for the prediction of ICI responsiveness using data extracted from The Cancer Genome Atlas (TCGA), Gene Expression Omnibus (GEO), and METABRIC databases. We combined these data with those in the immunology database and analysis portal ImmPort to investigate the relevant molecular mechanisms and immune cell relationships. Furthermore, from the breast cancer database of Peking Union Medical College Hospital, we identified the relationships between IGF2R expression and the clinical characteristics of TILs.

## Materials and methods

### Collection of breast cancer gene expression data

This was a retrospective study of the gene expression and the corresponding clinical data of patients included in two independent datasets obtained from publicly available databases. In total, the data from 1,561 patients were analyzed. The expression of 56,737 genes and the survival outcome data of 1,053 patients were obtained from TCGA (https://portal.gdc.cancer.gov/repository). Data on gene expression and DFS of 508 patients were retrieved from the GEO database (https://www.ncbi.nlm.nih.gov/geo/query/acc.cgi?acc=GSE25066). The expression of the IGF2R gene and the clinical characteristics and survival outcomes of 1,818 patients were acquired from the METABRIC database (http://www.cbioportal.org/datasets).

### Construction of the prognostic model based on immune-related gene pairs

To construct a prognostic model based on immune-related genes, 2,498 immune-related genes were obtained from the ImmPort database (https://www.immport.org/home) on 30 May 2020. This gene platform includes a list of immunologically relevant genes, curated with functions and Gene Ontology (GO) terms. The ImmuneRegulation web-based tool identified regulators of immune system-specific genes of interest, and the Immcantation framework analyzed high-throughput adaptive immune receptor repertoire sequencing datasets characterizing B-cell and T-cell receptors. In this study, we retained only immune-related genes that were identified in both the GEO and TCGA datasets with a median absolute deviation of >0.5 (24). The relative expression within each immune-related gene pair was compared for each patient in the TCGA dataset. In each pair, if the expression of one gene was larger than that of the other, then the value of the gene pair was considered to be 1; otherwise, the value was considered to be 0. After removing immune-related gene pairs with relatively small variations in expression within the pair (<20%), least absolute shrinkage and selection operator (Lasso) regression was performed for 1,000 simulations, and a prognostic model containing 26 immune-related gene pairs was obtained. This model was used to calculate the risk value of each patient in the TCGA dataset. A receiver operating characteristic curve was established using the risk values, and an optimal cutoff value was determined to distinguish between the low- and high-risk patients.

### Verification of the prognostic model based on immune-related gene pairs

To further verify the prognostic model based on immune-related gene pairs, the GEO dataset was used as the validation group. The risk value of each patient in the GEO dataset was calculated using the model, and the cutoff value obtained for the training group was used to stratify the GEO patients into high- and low-risk groups. Univariate and multivariate Cox proportional hazards analyses were used to verify whether the model could be used as an independent prognostic factor relative to other clinical features such as age, HR expression, HER2 expression, and American Joint Committee on Cancer (AJCC) stage in the GEO and TCGA datasets.

### Immune cell infiltration is associated with the prognostic model based on immune-related gene pairs

The CIBERSORT algorithm was used to estimate differences in immune cell infiltration using gene expression data in the high- and low-risk TCGA groups (25). This algorithm uses gene expression data to predict the proportions of 22 types of tumor-infiltrating immune cells, such as T cells, B cells, macrophages, and natural killer cells.

### Enrichment analysis by GO

Enrichment analysis of the identified immune-related genes was performed using g:Profiler (26). All GO gene sets were downloaded from the Gene Set Enrichment Analysis website (https://www.gsea-msigdb.org/gsea/index.jsp). Gene sets in the high- and low-risk TCGA groups were compared using the Bioconductor “fgsea” package in R. After 10,000 cycles, significant enrichment pathways were obtained and sequenced. Gene sets with statistical significance were selected with a false discovery rate–adjusted *P* < 0.05.

### Patients and immunohistochemistry

The tumor specimens from 282 patients with TNBC at stages I–III were collected. These patients received surgical treatment in our hospital between 2011 and 2014. Patients with stage IV TNBC and patients who received neoadjuvant chemotherapy were excluded. The formalin-fixed paraffin-embedded tumor specimens of these patients were made into tumor microarrays (TMAs). When constructing the TMAs, each tumor specimen included the epithelial components and the tumor stroma after hematoxylin and eosin (HE) staining. The median follow-up time was 69 months (1–104 months). All sections of the TMAs were stained with IGF2R, CD8, and CD19 antibodies, and the frequencies of TILs were evaluated according to the publication A Practical Review for Pathologists and Proposal (27). IGF2R expression was detected by a rabbit monoclonal antibody (#15128, Cell Signaling Technology; dilution, 1:50). CD8 expression was detected by 4B11 (PA0183, prediluted; Leica Microsystems, Shanghai, China). CD19 expression was detected by EP169 (ZA-0569, prediluted; Zhongshan Golden Bridge Biotechnology Co. Ltd., Beijing, China). Two pathologists reviewed all of the samples and scored the immunohistochemical staining independently. The expression of IGF2R was determined by histochemical scoring (H-score), with consideration of the staining intensity and the percentage of positive cancer cells (28). An H-score of 0–49 was classified as the negative group, whereas H-scores of 50–99, 100–199, and 200–300 were classified as 1+, 2+, and 3+, respectively. An H-score classification of 1+, 2+, or 3+ was classified as the positive group. TILs were divided into a low group and a high group, with a median of 5%. CD8+ TILs were also divided into a low group and a high group, with a median of 10%. Finally, CD19+ TILs were divided into a low group and a high group, with a median of 1%. Other pathological features of 282 patients, such as tumor stage, lymph node (LN), tumor grade, and Ki-67, were retrieved from the pathological report of the Beijing Union Medical College Hospital.

### Statistical analysis

Statistical analyses were performed using version 3.6.3 of the R Statistical Software and SPSS 23.0. Comparisons of genes between groups were performed using a t-test. The Kaplan–Meier method and the “survival” package in R were used for survival analysis. Cox proportional hazards regression analysis was used for univariate and multivariate analyses of OS or DFS. The Wilcoxon test was used to compare differences in immune cell infiltration. The chi-squared test was used to compare TILs, CD8+ TILs, and CD19+ TILs, and P < 0.05 was considered statistically significant. Statistical differences were recorded as follows: *P < 0.05, **P < 0.01, and ***P < 0.001.

## Results

### Construction of the prognostic model based on immune-related gene pairs

A total of 56,735 genes and 2,498 unique immune-related genes were obtained from the TCGA and ImmPort databases, respectively. Among them, 1,653 immune-related genes were included in the data obtained from both databases. Then, the immune-related genes from ImmPort and the genes obtained from the GEO dataset were intersected to locate the same genes. Among 606 common immune-related genes, 31,896 immune-related gene pairs were found after removing gene pairs with relatively small internal variations. The immune-related gene pairs from the TCGA dataset were combined with the corresponding clinical data, revealing 69 immune-related gene pairs that were significantly associated with the patient prognosis. Next, the Lasso method for Cox proportional hazards regression analysis was used to construct the prognostic model based on immune-related gene pairs for the training group. Finally, 26 immune-related gene pairs comprising 43 immune-related genes were selected in the model (**Table 1**).

**Table 1:** Prognostic model based on immune-related gene pairs.

IRG1	IRG2	Coefficient
IGF1R	IGF2R	-0.280680737393679
CD74	CRABP2	-0.129381388033907
HSPA2	NEDD4	-0.431472955510512
CIITA	TLR7	-0.0861166676605254
CIITA	PLXNB3	-0.1318512759267
MICA	PLXNB1	0.397483055209339
RELB	CCR1	-0.126764197388246
RFXAP	IGLV6-57	0.0399268657548744
TAPBPL	IGF2R	-0.266631594480481
CXCL14	HMOX1	-0.0424869848483334
CCL8	CD3D	0.123645099256706
S100B	PLXNB3	-0.0745978715504017
APOBEC3G	PLXNB3	-0.349449105911909
TRIM5	IL27RA	0.187761415761893
TYK2	PTK2	-0.237158486425192
MSR1	|IL18	0.2957793442697
PPARG	PLXNB3	-0.128237814963708
VAV1	ITGAL	0.318667102339933
RAC2	C3AR1	-0.319397161742718
IGHD	BTC	-0.00256266119415776
IGHD	SCG2	-0.019365921132164
IGHD	NPR3	-0.252893524646672
IGHD	ZAP70	-0.194457565172438
SEMA3B	SEMA6C	-0.192591720762394
SEMA3B	BTC	-0.18492459694902
ACVRL1	IL27RA	0.116702510407455

### Prognostic value of the model based on immune-related gene pairs for survival analysis

**Figure 1:**
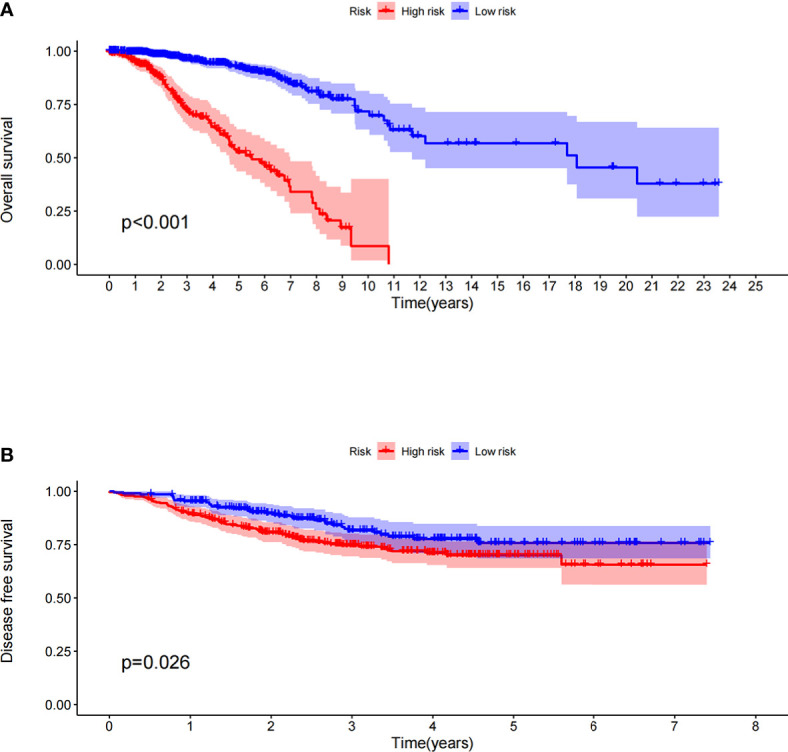
**(A)** Overall survival between the high- and low-risk patients in the TCGA datasets. **(B)** Disease-free survival between the high- and low-risk patients in the GEO datasets.

The prognostic model significantly distinguished between high- and low-risk patients in terms of their OS in the TCGA dataset; the OS of the high-risk patients was significantly shorter than that of the low-risk patients (**Figure 1A**, *P <* 0.001). To verify the predictive value of the prognostic model based on immune-related gene pairs, we applied the model to the GEO dataset and stratified the patients into high- and low-risk groups. The DFS values of the two validation groups were similar to those of the training groups; the DFS of the high-risk group was significantly less than that of the low-risk group (**Figure 1B**, *P* = 0.026). Next, univariate and multivariate Cox proportional hazards regression analyses were used to study the corresponding clinical data in the TCGA dataset. The prognostic model based on immune-related gene pairs and the AJCC stage were determined to be independent prognostic factors in the TCGA dataset (**Supplementary Figures 1A, B**). In addition, univariate and multivariate Cox proportional hazards regression analyses were used to analyze the corresponding clinical data in the GEO dataset (**Supplementary Figures 1C, D**). The prognostic model based on immune-related gene pairs was determined to be an independent prognostic factor in the GEO dataset, according to the univariate Cox proportional hazards regression analysis (**Supplementary Figure 1C**).

### Immune cell infiltration in different risk groups

CIBERSORT, which has been applied to many tumor microenvironments (29), was used to predict the infiltration of 21 different immune cell types in the high- and low-risk TCGA groups (**Supplementary Figure 2A**), including M0 and M2 macrophages, CD8^+^ T cells, and resting dendritic cells. M0 (**Supplementary Figure 2B**, *P* < 0.001) and M2 (**Supplementary Figure 2C**, *P* < 0.001) macrophages were highly expressed in the high-risk group, whereas CD8^+^ T cells (**Supplementary Figure 2D**, *P* < 0.001) and naive B cells (**Supplementary Figure 2E**, *P* < 0.001) were highly expressed in the low-risk group.

### Functional evaluation of immune-related gene pairs

To determine the biological processes and signaling pathways associated with the immune-related gene pairs in the prognostic model, GO enrichment was used to analyze the identified immune-related genes in the TCGA dataset, and pathways with significant differences between high- and low-risk patients were detected (**Supplementary Figure 3**). CCR chemokine receptor binding, regulation of leukocyte-mediated cytotoxicity, T-cell migration, T-cell receptor complex, and other pathways were determined to be significantly enriched in low-risk patients (**Supplementary Figures 4A–F**). The enrichment of these pathways in low-risk patients confirmed the importance of immune cells in the treatment and prognosis of patients with breast cancer.

### Prognostic value of IGF2R and its relationship with clinical characteristics

**Figure 2:**
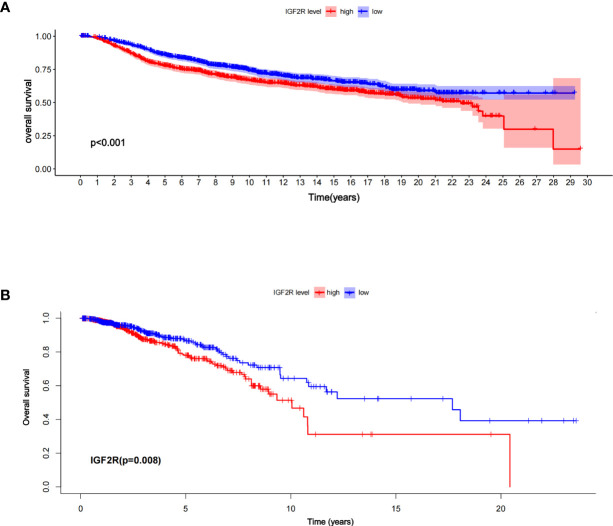
Overall survival between the high- and low-risk patients in **(A)** the TCGA datasets and **(B)** the METABRIC, according to IGF2R expression.

**Figure 3:**
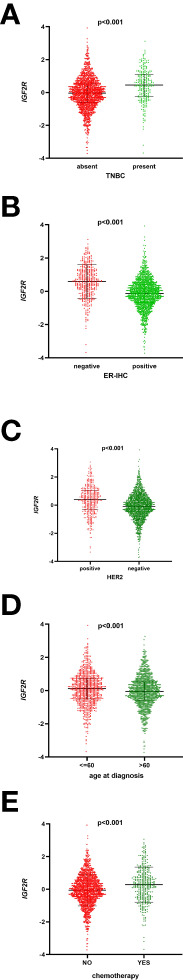
IGF2R expression with other clinical characteristics in the METABRIC datasets. **(A)** IGF2R expression in patients with TNBC and in other patients. **(B)** IGF2R expression in ER-negative patients and ER-positive patients. **(C)** IGF2R expression in HER2-positive patients and HER2-negative patients. **(D)** IGF2R expression in patients aged ≤60 years old and in patients aged >60 years old. **(E)** IGF2R expression in patients with chemotherapy and in patients without chemotherapy.

Of the 1,098 patients with breast cancer in the TCGA dataset, those with a higher level of IGF2R had a lower OS compared with those with a lower level of IGF2R (**Figure 2A**, *P <* 0.001). In the METABRIC dataset including 1,818 patients with breast cancer, the OS was lower in the patients with a higher IGF2R expression level compared with the patients with a lower IGF2R expression level (**Figure 2B**, P = 0.008). Of the patients included in the METABRIC dataset, IGF2R expression was greater in the patients with TNBC vs. the other patients (**Figure 3A**, P < 0.001), in the ER-negative patients vs. the ER-positive patients (**Figure 3B**, P < 0.001), in the HER2-positive patients vs. the HER2-negative patients (**Figure 3C**, P < 0.001), in the patients aged ≤60 years old vs. those aged >60 years old (**Figure 3D**, P < 0.001), and in patients that had undergone chemotherapy vs. those without chemotherapy (**Figure 3E**, P < 0.001).

### Prognostic value of IGF2R and its relationship with clinical characteristics in patients with TNBC

**Figure 4:**
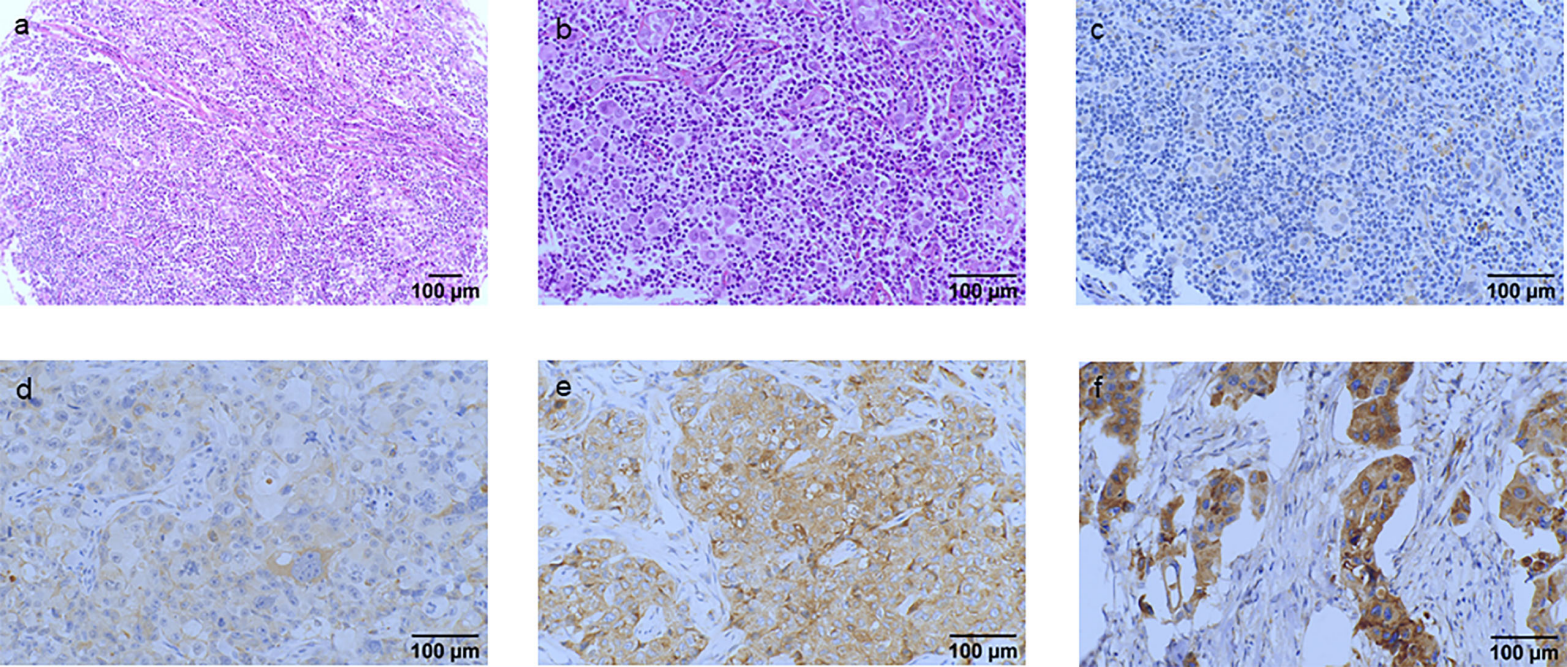
Hematoxylin and eosin (HE) staining and classification of IGF2R expression by the H-score in patients with TNBC. **(A)** HE staining of the tumor cells (original magnification, ×100). **(B)** HE staining of the tumor cells (original magnification, ×200). **(C)** IGF2R-negative expression on tumor cells (original magnification, ×200). **(D)** IGF2R 1+ expression on tumor cells (original magnification, ×200). **(E)** IGF2R 2+ expression on tumor cells (original magnification, ×200). **(F)** IGF2R 3+ expression on tumor cells (original magnification, ×200).

**Figure 5:**
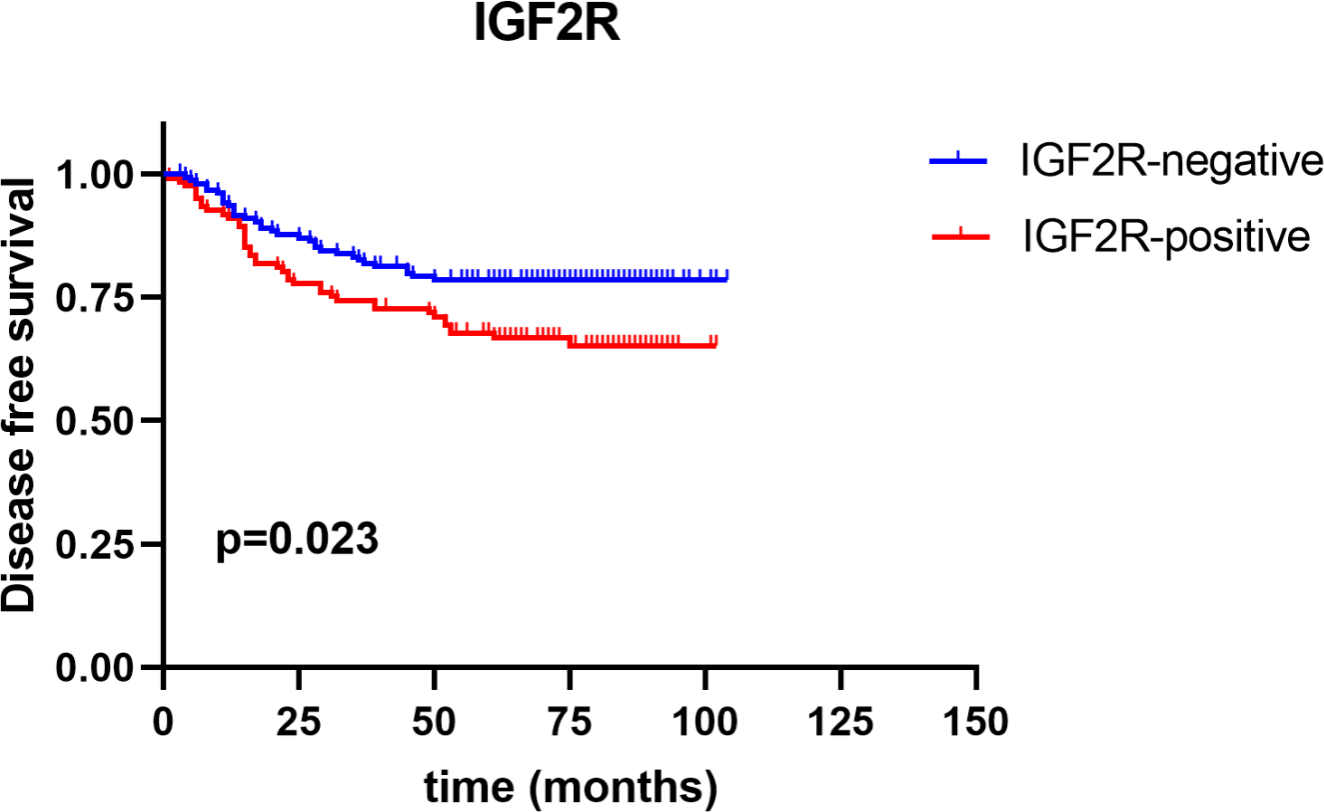
Disease-free survival between IGF2R-positive patients and IGF2R-negative patients in the TNBC cohort.

**Figure 6:**
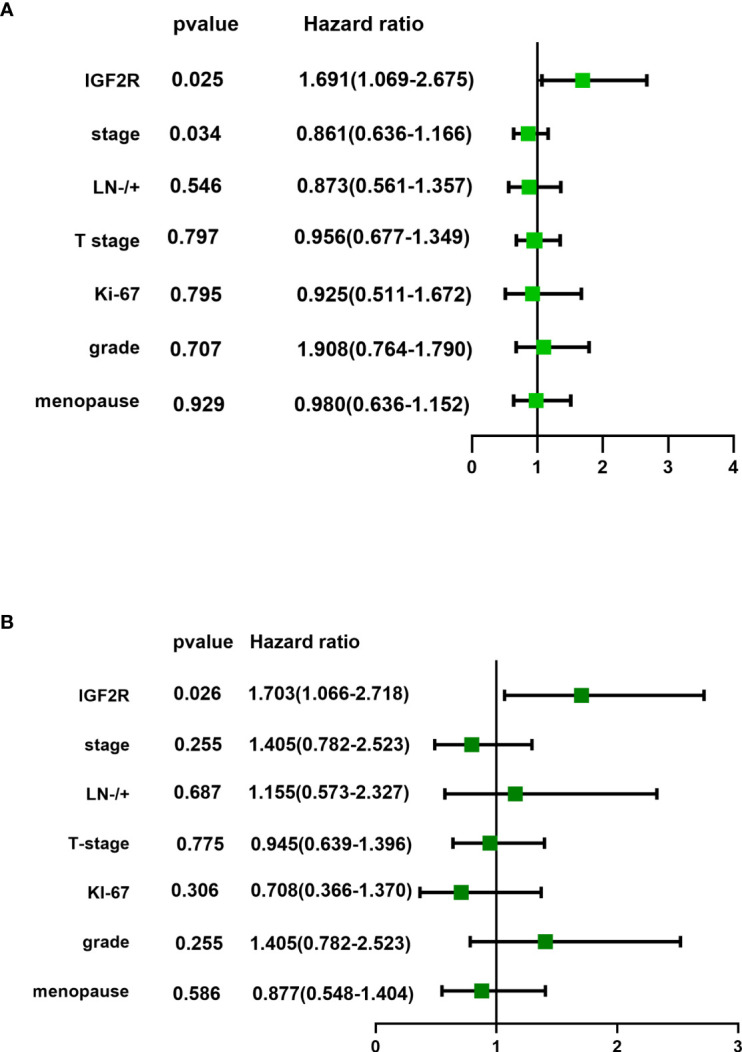
**(A)** Univariate analysis and **(B)** multivariate analysis of IFG2R expression and clinical characteristics for patients in the TNBC cohort.

IGF2R staining was evaluated in a cohort of 282 patients with TNBC. There were 159 (56.4%) patients with an H-score of 0, 63 (22.3%) patients with an H-score of 1+, 54 (19.1%) patients with an H-score of 2+, and six (2.1%) patients with an H-score of 3+ (**Figure 4**). The 4-year DFS of IGF2R-positive TNBC patients was lower compared with that of IGF2R-negative patients (**Figure 5**; 67.8% vs. 78.5%, *P* = 0.023). IGF2R expression independently predicted the DFS in univariate and multivariate Cox proportional hazards regression analyses (**Figure 6A**, *P* = 0.025; **Figure 6B**, *P* = 0.026), whereas the AJCC stage, LN−/+, T-stage, Ki-67, grade, and menopause status of patients did not predict the DFS.

### Prognostic value of IGF2R and immune markers in the tumor microenvironment of TNBC

**Figure 7:**
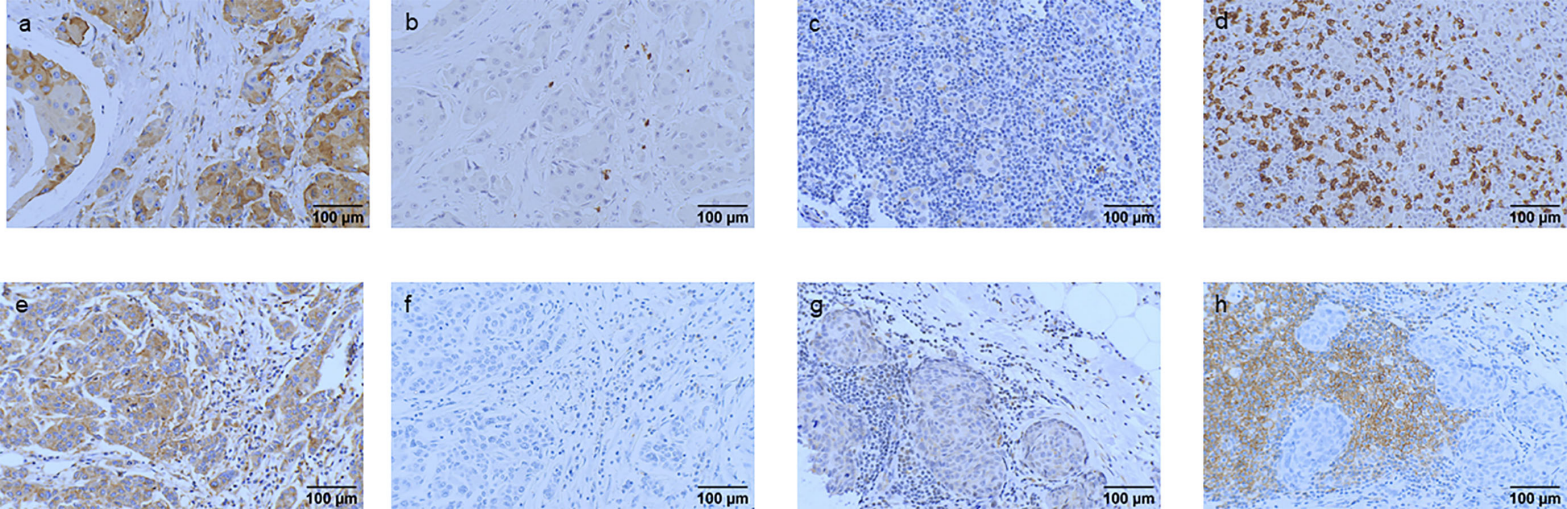
IGF2R expression association with CD8^+^ TILs and CD19^+^ TILs. **(A, B)** The patients with IGF2R-positive expression and a low percentage of CD8^+^ TILs. **(C, D)** The patients with IGF2R-negative expression and a high percentage of CD8^+^ TILs. **(E, F)** The patients with IGF2R-positive expression and a low percentage of CD19^+^ TILs. **(G, H)** The patients with IGF2R-negative expression and a high percentage of CD19^+^ TILs.

**Figure 8:**
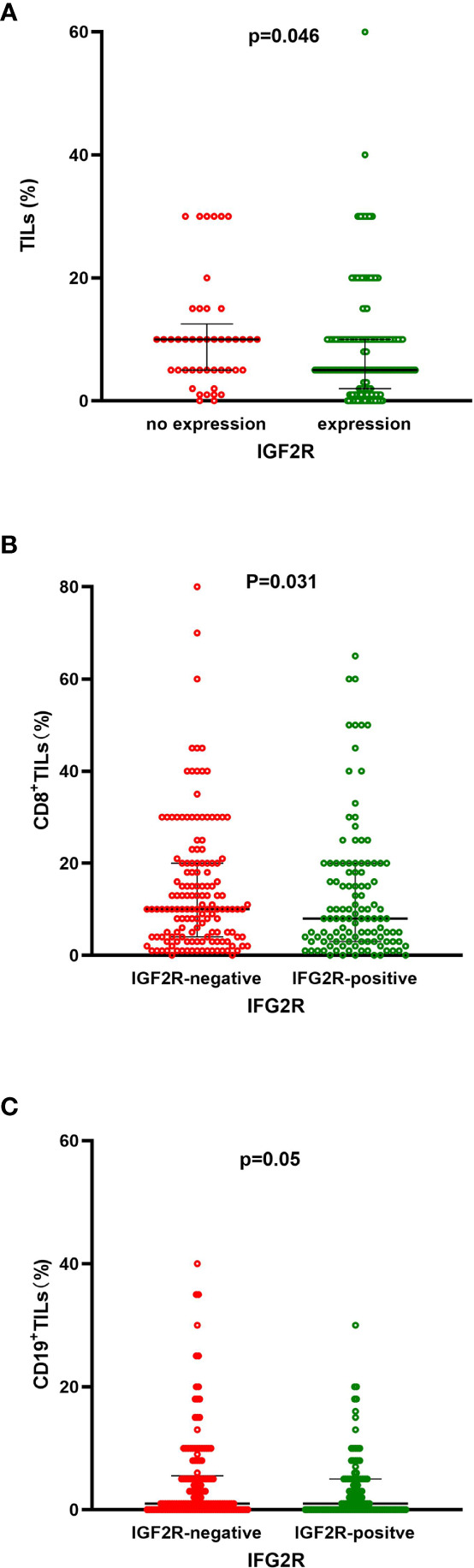
**(A)** Frequencies of TILs between IGF2R-negative expression and IGF2R-positive expression patients in the TNBC cohort. **(B)** Frequencies of CD8^+^ TILs between IGF2R-negative expression and IGF2R-positive expression patients in the TNBC cohort. **(C)** Frequencies of CD19^+^ TILs between IGF2R-negative expression and IGF2R-positive expression patients in the TNBC cohort.

TILs, CD8^+^ TILs, and CD19^+^ TILs were detected in the 282 patients with TNBC. High frequencies of TILs, CD8^+^ TILs, and CD19^+^ TILs were observed in 97.0%, 44.3%, and 63.5% of patients with TNBC. The percentages of TILs, CD8^+^ TILs, CD19^+^ TILs, and IGF2R expression were analyzed (**Figure 7**). Patients with TNBC with IGF2R-positive expression had lower frequencies of TILs compared with patients with IGF2R-negative expression (**Figure 8A**, *P* = 0.046). Moreover, patients with TNBC with IGF2R-positive expression had lower densities of CD8^+^ TILs and CD19+ TILs compared with those with IGF2R-negative expression (**Figures 8B, C**, *P* = 0.031 and *P* = 0.05).

## Discussion

In the current study, we demonstrated that a prognostic model constructed with 26 immune-related gene pairs from 43 independent immune-related genes predicted the OS and DFS of patients with breast cancer. Relative sequencing and pairing of genes to create a prognostic model based on immune-related gene pairs have provided reliable results for many types of tumors (30, 31). The prognostic model based on immune-related gene pairs was an independent prognostic factor in the TCGA dataset. The findings from this study revealed that M2 and M0 macrophages were highly expressed in high-risk patients with breast cancer, whereas CD8^+^ T cells and naive B cells were highly expressed in low-risk patients with breast cancer. In this study, the identified immune-related genes were associated with multiple pathways related to immune cell infiltration, migration, and immune checkpoint enhancement. Among them, regulation of the leukocyte-mediated cytotoxicity pathway is associated with tumor progression and decreased CD8^+^ infiltration in pancreatic cancer (32). In addition, the T-cell migration pathway has been demonstrated to enhance tumor immunity and increase the efficacy of ICIs in preclinical breast cancer models (33). This pathway can increase the number of T cells in tumors and intratumoral T-cell diversity (34).

Among 43 independent immune-related genes, IGF2R was selected for further research. We found that a high level of IGF2R expression was significantly associated with a poor prognosis in patients included in the TCGA and METABRIC databases. In addition, we observed that IGF2R was closely related with poor clinical characteristics, such as TNBC, premenopause, ER-negative expression, and chemotherapy. Furthermore, we identified that the expression level of IGF2R was significantly associated with a poor prognosis and lower frequencies of TILs and CD8+ TILs in the cohort of patients with TNBC.

IGF2R is a growth inhibitory factor 35. IGF2R deletion or mutation may contribute to the development and progression of cancer 36. The deletion of the IGF2R allele has been shown to be an early event in the etiology of breast cancer (36) as a tumor suppressor (37). In addition, low levels of IGF2R have been associated with a poor prognosis in patients with breast cancer (38). However, IGF2R is overexpressed in HR-negative breast cancer (39). Moreover, IGF2R plays a central role in the differentiation of TNBC subsets. The IGF receptor family also has been related to tumor differentiation and the prognosis of patients with TNBC (40). In fact, IGF2R has been demonstrated to be an unfavorable prognostic factor for patients with ER-negative breast cancer (41). In addition, luminal A and luminal B patients with a high expression of IGF1R and a low expression of IGF2R had significantly higher survival rates than patients with other types of breast cancer (39). Overexpression of IGF2R also has been shown to significantly increase the migration and invasion of MDA-MB-231 cells (42). The level of IGF2R mRNA in MDA-MB-231 cells has been determined to be higher than that in MCF-7 cells (43). Likewise, our study observed that a high expression of IGF2R was associated with a poor prognosis of patients with TNBC.

The development of breast cancer is characterized by an increased infiltration of immune cells in the parenchyma and stroma of a tumor (44). It has been demonstrated that stromal infiltrating lymphocytes (sTILs) have predictive and prognostic value for TNBC and that high percentages of sTILs indicate a better prognosis (44). CD8^+^ lymphocyte infiltration has been shown to be an independent favorable prognostic indicator in TNBC (45), and a high CD8^+^ T-cell score is associated with better survival rates in patients with TNBC (46). Improved outcomes of atezolizumab have been observed only in CD8^+^ and sTILs^+^ patients (47). The PARP inhibitor olaparib induces CD8^+^ T-cell infiltration through activation of the cyclic GMP-AMP synthase/stimulator of interferon genes pathway, and CD8^+^ T-cell depletion severely compromises antitumor efficacy (48). Immunotherapy methods, including PD-1/PD-L1 blocking and chimeric antigen receptor T-cell therapy, have been shown to improve antitumor activity through the proliferation of CD8 ^+^ T cells (49). Although PD-1/PD-L1 blocking has triggered great progress in the treatment of TNBC, the benefits are still limited (50–52). Therefore, more immunological targets and therapies through TILs need to be explored to improve the survival rates of patients with TNBC.

The IGF2R polyclonal antibody has been demonstrated to induce the blockade of T-cell differentiation at the CD8^−^ stage and decrease the percentage of CD8^+^ cells (53). In addition, it has been shown that IGF2R and CD8^+^ T cells coexist in transplanted hearts and are involved in acute cellular rejection (54). Moreover, the enhancement of IGF2R expression has been revealed to increase apoptosis in CD8α+ dendritic cells, with a consequent reduction in the expression of interleukin (IL)-12 and interferon (IFN)-γ (55), while IL-12 and IFN-α/β provide signal support for CD8+ T memory programming (56). Furthermore, IGF2R is involved in the activation of TGFβ (57). TGFβ enhancement inhibits the proliferation of regulatory T cells (58), decreases the CD8+ T effector cell penetration in tumors (59), and suppresses the immune response (60). This may explain our findings, which show that patients with TNBC with a high expression of IGF2R had a low density of TILs and a low density of CD8^+^ TILs, thus promoting immune escape and leading to a poor prognosis for these patients.

CD19 is the common therapeutic target of hematological malignancies (61). Anti-CD19 chimeric antigen receptor T-cell therapy directed against B-cell lymphoma has been demonstrated to be efficacious. However, efforts to utilize this approach for breast cancer have delivered only modest improvements (62). The frequencies of CD19+ B cells in breast cancer are greater than those in normal tissues (63). Compared with fibroadenoma, the density of CD19+ B cells in breast cancer is greater and is significantly associated with higher tumor grades and an ER-negative status (64). CD19 is also highly expressed in patients with high-risk breast cancer (65). CD19+ B lymphocytes play an important role in breast cancer through PD-L1 in immune suppression and tumor escape (64). CD19+CD25+ regulatory B cells inhibit TILs and are closely related to the metastasis of breast cancer (66). These studies suggest that CD19^+^ B cells are a feature of patients with breast cancer with a poor prognosis. In our research, we found that a high percentage of CD19^+^ TILs was associated with a low expression of IGF2R, whereas a low expression of IGF2R was correlated with a high percentage of TILs. Therefore, we speculate that IGF2R may cause breast cancer immune escape through CD19^+^ TILs. Future targeting of IGF2R may promote the proliferation of CD19^+^ TILs by reducing the expression of inflammatory factors, thereby inhibiting the progression of TNBC.

IGF2R inhibits the proliferation of T cells and the infiltration of T cells in a tumor by TGFβ activation. In addition, IGF2R has been shown to promote the secretion of IL-10 by B cells (67), and IL-10 directly activates or expands T cells in a tumor (68). However, IL-10 promotes the depletion of CD8^+^ T cells *in vivo (* 69) and inhibits the activity of CD8^+^ T cells (70). The invasion of IL-10 has been demonstrated to activate CD19^+^ B cells in the pathogen (71). Whether or not IGF2R inhibits the recruitment of TILs through some pathways or cytokines and whether it promotes the depletion of TILs in the tumor might be a direction for future research related to TNBC. An IGF2R inhibitor might be one of the target drugs for the treatment of TNBC in the future.

In summary, by analyzing patients from the TCGA and GEO databases, we found that IGF2R is a gene that is associated with a poor prognosis in patients with breast cancer. In the METABRIC database, the expression of IGF2R distinguished patients with breast cancer with a poor prognosis from those with a more favorable prognosis and was highly expressed in patients with TNBC. Patients with a high expression of IGF2R had a poor prognosis, and a high IGF2R expression was negatively correlated with TILs, CD8^+^ TILs, and CD19^+^ TILs in the TNBC cohort. The datasets originated from retrospective studies including patients without ICI therapies. Thus, our prognostic model and IGF2R expression must be more widely validated in prospective cohort studies. In addition, the association of IGF2R, CD8^+^ TILs, and CD19^+^ TILs as well as their related immune factors and molecular mechanisms require further verification *in vitro* and *in vivo*.

## Conclusion

We established a prognostic model based on immunogenomics that reliably predicted the prognosis of patients with breast cancer. We identified that the immune-related gene IGF2R may play an important role in the treatment of TNBC in the future and may provide new targets for immunotherapy. Furthermore, we demonstrated that CD8^+^ TILs and CD19^+^ TILs were highly expressed in patients with TNBC with a low expression of IGF2R. Targeting CD8^+^ TILs and CD19^+^ TILs combined with IGF2R expression should be investigated in future TNBC treatment research.

## Data availability statement

The datasets presented in this study can be found in online repositories. The names of the repository/repositories and accession number(s) can be found in the article/**Supplementary Material**.

## Ethics statement

The studies involving human participants were reviewed and approved by Human Research of Peking Union Medical College Hospital. The patients/participants provided their written informed consent to participate in this study. Written informed consent was obtained from the individual(s) for the publication of any potentially identifiable images or data included in this article.

## Author contributions

YZ collected and analyzed clinical and gene data. Experiments were performed by YZ, XR, and XC. YX, YDZ, FM, SS, and ZW collected and evaluated the TNBC cohort. YZ, YS, and ZW conducted statistical analysis. QS supported quality management and directed the team. All authors contributed to the article and approved the submitted version.

## Funding

This study was supported by the CAMS Innovation Fund for Medical Sciences (CIFMS) (2021-I2M-C&T-B-017).

## Acknowledgments

The authors would like to thank the TCGA, GEO, GO, and METABRIC databases and the Department of Pathology of Peking Union Medical College Hospital for the availability of the data. The authors would also like to thank all of the patients who participated in this study.

## Conflict of interest

The authors declare that the research was conducted in the absence of any commercial or financial relationships that could be construed as a potential conflict of interest.

## Publisher’s note

All claims expressed in this article are solely those of the authors and do not necessarily represent those of their affiliated organizations, or those of the publisher, the editors and the reviewers. Any product that may be evaluated in this article, or claim that may be made by its manufacturer, is not guaranteed or endorsed by the publisher.

## Supplementary material

Supplementary Figure 1
**(A)** Univariate analysis and **(B)** multivariate analysis of the prognostic model based on immune-related gene pairs for patients in the TCGA datasets. **(C)** Univariate analysis and **(D)** multivariate analysis of the prognostic model based on immune-related gene pairs for patients in the GEO datasets.

Supplementary Figure 2
**(A)** Infiltration of 21 different immune cell types, **(B)** M0 macrophage infiltration, **(C)** M2 macrophage infiltration, **(D)** CD8^+^ T cell infiltration, and **(E)** naive B cell infiltration in the high- and low-risk groups by the prognostic model based on immune-related gene pairs in the TCGA datasets.

Supplementary Figure 3 Pathways identified with significant differences between high- and low-risk groups by the prognostic model based on immune-related gene pairs in the TCGA datasets.

Supplementary Figure 4
**(A)** The CCR chemokine receptor binding pathway was significantly enriched in low-risk patients by the prognostic model based on immune-related gene pairs. **(B)** The regulation of the leukocyte-mediated cytotoxicity pathway was significantly enriched in low-risk patients by the prognostic model based on immune-related gene pairs. **(C)** The T cell migration pathway was significantly enriched in low-risk patients by the prognostic model based on immune-related gene pairs. **(D)** The T cell receptor complex pathway was significantly enriched in low-risk patients by the prognostic model based on immune-related gene pairs. **(E)** The cytokine receptor binding pathway was significantly enriched in low-risk patients by the prognostic model based on immune-related gene pairs. **(F)** The response to the chemokine pathway was significantly enriched in low-risk patients by the prognostic model based on immune-related gene pairs.
