# Supplementary material for: Insulin-like growth factor 2 receptor is a key immune-related gene that is correlated with a poor prognosis in patients with triple-negative breast cancer: A bioinformatics analysis
Source: Front Oncol. 2022 Oct 18;12:871786. doi: 10.3389/fonc.2022.871786 (PMC9624382; doi:10.3389/fonc.2022.871786)
Supplement: Supplementary Figure 1 — (A) Univariate analysis and (B) multivariate analysis of the prognostic model based on immune-related gene pairs for patients in the TCGA datasets. (C) Univariate analysis and (D) multivariate analysis of the prognostic model based on immune-related gene pairs for patients in the GEO datasets. [file Presentation_1.pdf]

a

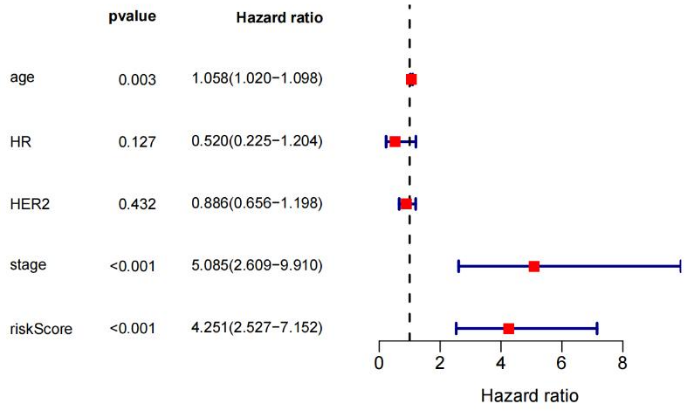

pvalue
Hazard ratio
age 0.003 1.058(1.020−1.098)
HR 0.127 0.520(0.225−1.204)
HER2 0.432 0.886(0.656−1.198)
stage <0.001 5.085(2.609−9.910)
riskScore <0.001 4.251(2.527−7.152)
0 2 4 6 8
Hazard ratio

b

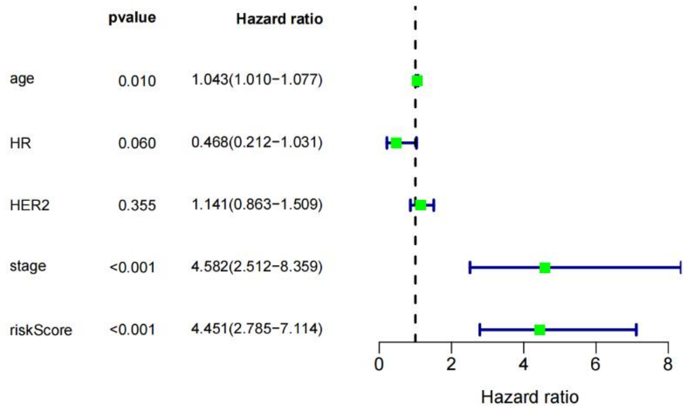

pvalue
Hazard ratio
age 0.010 1.043(1.010−1.077)
HR 0.060 0.468(0.212−1.031)
HER2 0.355 1.141(0.863−1.509)
stage <0.001 4.582(2.512−8.359)
riskScore <0.001 4.451(2.785−7.114)
0 2 4 6 8
Hazard ratio

c

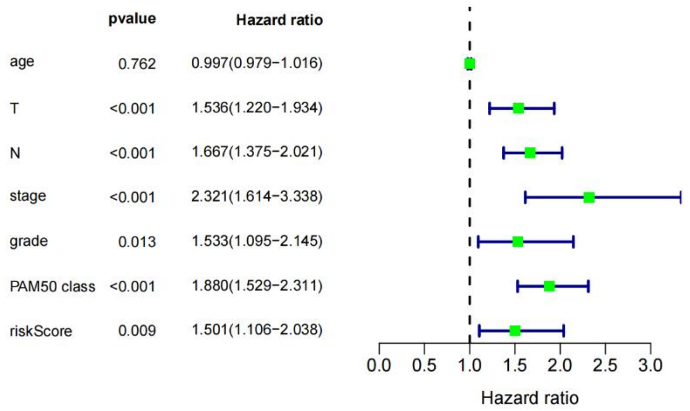

pvalue
Hazard ratio
age 0.762 0.997(0.979−1.016)
T <0.001 1.536(1.220−1.934)
N <0.001 1.667(1.375−2.021)
stage <0.001 2.321(1.614−3.338)
grade 0.013 1.533(1.095−2.145)
PAM50 class <0.001 1.880(1.529−2.311)
riskScore 0.009 1.501(1.106−2.038)
0.0 0.5 1.0 1.5 2.0 2.5 3.0
Hazard ratio

d

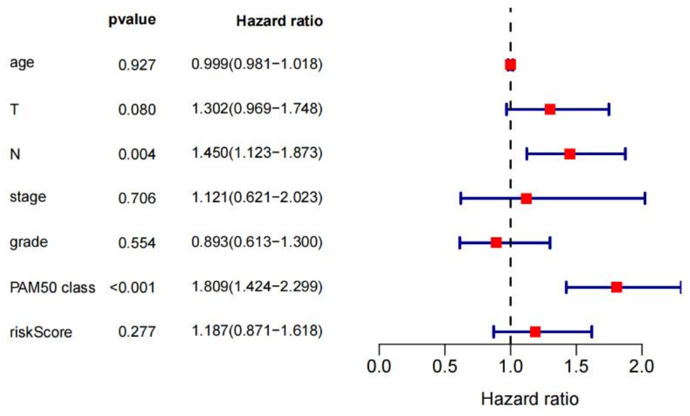

pvalue
Hazard ratio
age 0.927 0.999(0.981−1.018)
T 0.080 1.302(0.969−1.748)
N 0.004 1.450(1.123−1.873)
stage 0.706 1.121(0.621−2.023)
grade 0.554 0.893(0.613−1.300)
PAM50 class <0.001 1.809(1.424−2.299)
riskScore 0.277 1.187(0.871−1.618)
0.0 0.5 1.0 1.5 2.0
Hazard ratio

SUPPLEMENTARY FIGURE 1 (a) Univariate analysis and (b) multivariate analysis of the prognostic model based on immune-related gene pairs for patients in the TCGA datasets. (c) Univariate analysis and (d) multivariate analysis of the prognostic model based on immune-related gene pairs for patients in the GEO datasets.

a

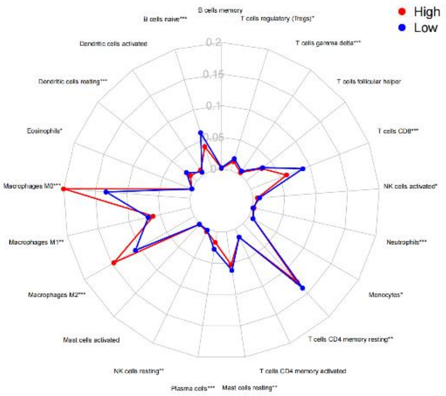
High
Low
B cells memory
T cells regulatory (Tregs)*
T cells gamma delta***
T cells follicular helper
T cells CD8***
NK cells activated*
Neutrophils***
Monocytes*
T cells CD4 memory resting**
T cells CD4 memory activated
Mast cells resting**
Plasma cells***
NK cells resting**
Mast cells activated
Macrophages M2***
Macrophages M1**
Macrophages M0***
Eosinophils*
Dendritic cells resting***
Dendritic cells activated
B cells naive***
0.2
0.15
0.1
0.05
0

b

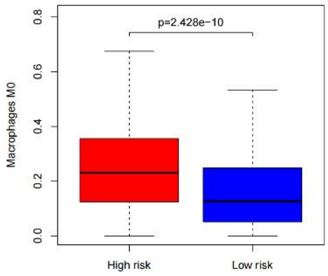
p=2.428e−10
Macrophages M0
0.0
0.2
0.4
0.6
0.8
High risk
Low risk

c

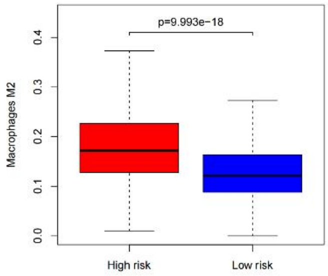
p=9.993e−18
Macrophages M2
0.0
0.1
0.2
0.3
0.4
High risk
Low risk

d

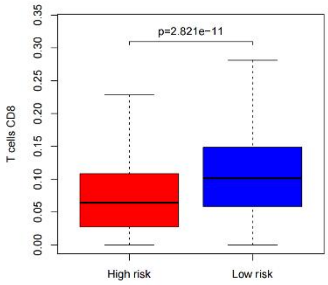
p=2.821e−11
T cells CD8
0.00
0.05
0.10
0.15
0.20
0.25
0.30
0.35
High risk
Low risk

e

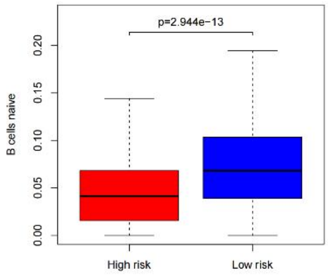
p=2.944e−13
B cells naive
0.00
0.05
0.10
0.15
0.20
High risk
Low risk

SUPPLEMENTARY FIGURE 2 (a) Infiltration of 21 different immune cell types, (b) M0 macrophage infiltration, (c) M2 macrophage infiltration, (d) $CD8^{+}$ T cell infiltration, and (e) naive B cell infiltration in the high- and low-risk groups by the prognostic model based on immune-related gene pairs in the TCGA datasets.

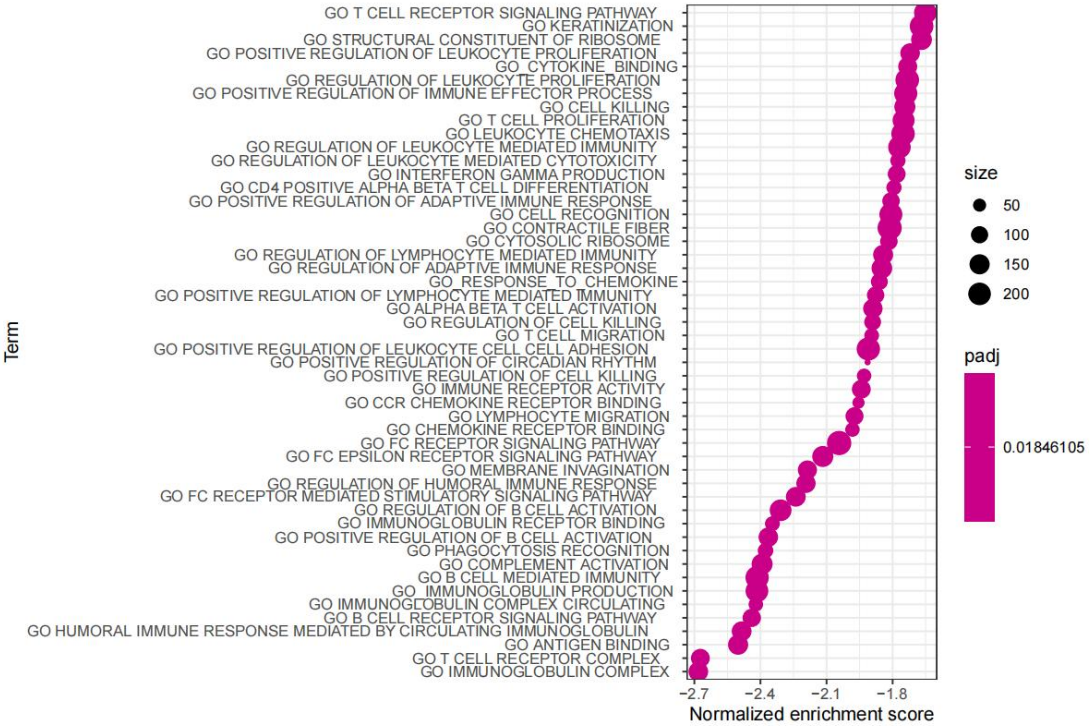

SUPPLEMENTARY FIGURE 3 Pathways identified with significant differences between high- and low-risk groups by the prognostic model based on immune-related gene pairs in the TCGA datasets.

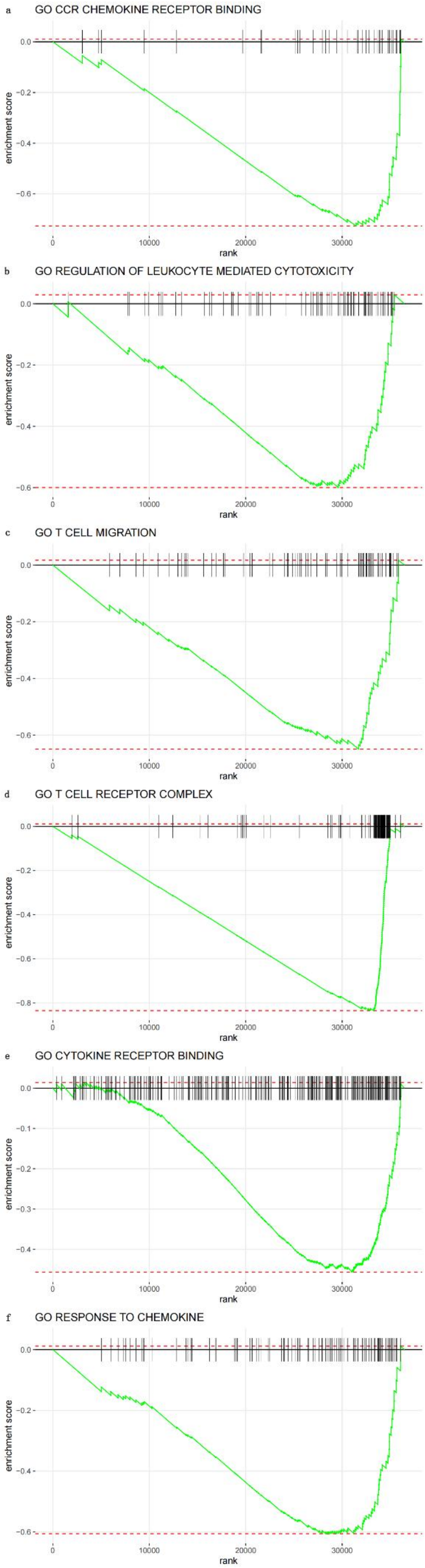

a
GO CCR CHEMOKINE RECEPTOR BINDING
enrichment score
0.0
-0.2
-0.4
-0.6
0
10000
20000
30000
rank
b
GO REGULATION OF LEUKOCYTE MEDIATED CYTOTOXICITY
enrichment score
0.0
-0.2
-0.4
-0.6
0
10000
20000
30000
rank
c
GO T CELL MIGRATION
enrichment score
0.0
-0.2
-0.4
-0.6
0
10000
20000
30000
rank
d
GO T CELL RECEPTOR COMPLEX
enrichment score
0.0
-0.2
-0.4
-0.6
-0.8
0
10000
20000
30000
rank
e
GO CYTOKINE RECEPTOR BINDING
enrichment score
0.0
-0.1
-0.2
-0.3
-0.4
0
10000
20000
30000
rank
f
GO RESPONSE TO CHEMOKINE
enrichment score
0.0
-0.2
-0.4
-0.6
0
10000
20000
30000
rank

SUPPLEMENTARY FIGURE 4 (a) The CCR chemokine receptor binding pathway was significantly enriched in low-risk patients by the prognostic model based on immune-related gene pairs. (b) The regulation of the leukocyte-mediated cytotoxicity pathway was significantly enriched in low-risk patients by the prognostic model based on immune-related gene pairs. (c) The T cell migration pathway was significantly enriched in low-risk patients by the prognostic model based on immune-related gene pairs. (d) The T cell receptor complex pathway was significantly enriched in low-risk patients by the prognostic model based on immune-related gene pairs. (e) The cytokine receptor binding pathway was significantly enriched in low-risk patients by the prognostic model based on immune-related gene pairs. (f) The response to the chemokine pathway was significantly enriched in low-risk patients by the prognostic model based on immune-related gene pairs.
